# Regulation of aggregate size and pattern by adenosine and caffeine in cellular slime molds

**DOI:** 10.1186/1471-213X-12-5

**Published:** 2012-01-23

**Authors:** Pundrik Jaiswal, Thierry Soldati, Sascha Thewes, Ramamurthy Baskar

**Affiliations:** 1 Department of Biotechnology, Indian Institute of Technology-Madras, Chennai-600036, India; 2 Département de Biochimie, Faculté des Sciences, Université de Genève, Sciences II, 30 quai Ernest Ansermet, CH-1211 Genève-4, Switzerland; 3 Institute for Biology-Microbiology; Department of Biology, Chemistry, Pharmacy; Freie Universität Berlin; D-14195 Berlin, Germany

## Abstract

**Background:**

Multicellularity in cellular slime molds is achieved by aggregation of several hundreds to thousands of cells. In the model slime mold *Dictyostelium discoideum*, adenosine is known to increase the aggregate size and its antagonist caffeine reduces the aggregate size. However, it is not clear if the actions of adenosine and caffeine are evolutionarily conserved among other slime molds known to use structurally unrelated chemoattractants. We have examined how the known factors affecting aggregate size are modulated by adenosine and caffeine.

**Result:**

Adenosine and caffeine induced the formation of large and small aggregates respectively, in evolutionarily distinct slime molds known to use diverse chemoattractants for their aggregation. Due to its genetic tractability, we chose *D. discoideum *to further investigate the factors affecting aggregate size. The changes in aggregate size are caused by the effect of the compounds on several parameters such as cell number and size, cell-cell adhesion, cAMP signal relay and cell counting mechanisms. While some of the effects of these two compounds are opposite to each other, interestingly, both compounds increase the intracellular glucose level and strengthen cell-cell adhesion. These compounds also inhibit the synthesis of cAMP phosphodiesterase (PdsA), weakening the relay of extracellular cAMP signal. Adenosine as well as caffeine rescue mutants impaired in stream formation (*pde4^- ^*and *pdiA^-^*) and colony size (*smlA^- ^*and *ctnA^-^*) and restore their parental aggregate size.

**Conclusion:**

Adenosine increased the cell division timings thereby making large number of cells available for aggregation and also it marginally increased the cell size contributing to large aggregate size. Reduced cell division rates and decreased cell size in the presence of caffeine makes the aggregates smaller than controls. Both the compounds altered the speed of the chemotactic amoebae causing a variation in aggregate size. Our data strongly suggests that cytosolic glucose and extracellular cAMP levels are the other major determinants regulating aggregate size and pattern. Importantly, the aggregation process is conserved among different lineages of cellular slime molds despite using unrelated signalling molecules for aggregation.

## Background

During their life cycle, cellular slime molds alternate between unicellular and multicellular forms [1]. The unicellular amoebae feed on bacteria and retain their single cell identity as long as the food is abundant. At the onset of starvation, hundreds to hundreds of thousands of amoebae initiate a chemotactic signal-relay using polyketides, nucleotides or peptides and other unidentified signalling molecules to form a multicellular slug [2-7]. Cells at the anterior of the slug differentiate as a dead stalk while the rest of the cells encapsulate as spores in a fruiting body. Based on the small subunit ribosomal DNA (SSU) rDNA and α-tubulin amino acid sequences, the entire cellular slime mold 'Dictyostelia' are grouped in 4 distinct evolutionary lineages [8]. cAMP is a chemoattractant in all group 4 species including *D. discoideum, D. mucoroides and D. giganteum *[5,9] while in other groups at least three different compounds are used for aggregation. Group 3 species like *D. lacteum, D. minutum *and *D. tenue *make use of pterin, folic acid, and an unknown compound, respectively [3,4,7]. A modified dipeptide, glorin (N-propionyl-Y-L-glutamyl-L-ornithine and lactam ethyl ester) and an unknown compound act as chemoattractants in group 2 species, *Polysphondylium pallidum *and *P. luridum*, respectively [6]. It is not clear to what extent the signalling pathways that regulate aggregation are conserved between these different slime mold groups that use structurally unrelated chemoattractants.

The four major determinants known to regulate aggregate size in *D. discoideum *include the overall cell number and their size within the aggregate, the counting mechanism, cell-cell adhesion and cAMP signal strength [10,11].

The number and size of the individual cells within an organism determines its overall size or bulkiness [12,13], and signalling pathways that control cell growth such as the Target of Rapamycin (TOR) kinase pathway [14] and cell proliferation are important for controlling organ size. The number of cells required to form an aggregate of certain size is regulated by the counting mechanism that precisely counts and foretells when an aggregate of critical size is reached [15]. This is achieved by a set of secreted proteins, the concentration of which determines when an aggregate has to break or continue aggregation to reach certain size. In *D. discoideum*, the cell number available for aggregation is governed by a secreted factor called conditioned medium factor (CMF; [16,17]). The threshold concentration of CMF in the medium determines cAMP expression and secretion of countin factors by the amoebae [18,19]. The counting factor (CF) regulates intracellular glucose levels, cell movement and cell adhesion and maintains the integrity of aggregation streams [10,15]. CF is comprised of Countin, CF-50, CF-45-1, and CF-60 proteins, and increases cell movement by reducing the cytosolic glucose levels [10]. Indeed, cells starved in the presence of 1 mM glucose exhibit slower movement than untreated controls [10].

Cell-cell adhesion, another important determinant of aggregate size, is established through cell surface glycoproteins like Cad-1 (gp24) and CsaA (gp80) [15]. Cad-1 is a cadherin-dependent EDTA-sensitive glycoprotein, expressed in early stages of development and CsaA is an EDTA-resistant glycoprotein, expressed during later stages of development [20] the activation of which is cAMP dependent. The fourth factor known to affect aggregate size is the cAMP signal strength, which depends on cAMP-dependent adenyl cyclase activation, cAMP phosphodiesterase activity (PdsA and Pde4) and the concentration of the cAMP phosphodiesterase inhibitor (PDI) [21-24]. cAMP upon binding to its surface receptors (cAR1) activates adenyl cyclase to catalyze the conversion of ATP into cAMP [23]. The secreted cAMP further gets converted to 5'AMP by the action of PdsA, which is negatively regulated by a phospho diesterase inhibitor PDI [23]. Genetic lesions in the genes encoding for counting factor (Countin, CF-50, CF-45-1, CF-60), cell-cell adhesion (Cad-1, CsaA), or cAMP signal relay (Adenyl cyclase A (ACA), PdsA, cAR-1) and cAMP phosphodiesterase inhibitor (PDI), respectively, affect the aggregate size in *Dictyostelium *[23,25-29].

Adenosine, one of the morphogens identified in *D. discoideum *increases the aggregate size and influences cell fate during development [30]. It consists of adenine attached to a ribose sugar via a β-N9-glycosidic bond. It is a hydrolysed derivative of cAMP, synthesised within the slug tip, which represses competing tip initiation [30]. Pde4, an extracellular cAMP phosphodiesterase regulates cAMP levels in *Dictyostelium *slugs by catalysing the conversion of cAMP into 5'AMP [3], which further gets converted to adenosine by 5' nucleotidase [31]. The adenosine antagonist, caffeine represses signals that prevent tip formation, thereby inducing additional tips in *D. discoideum *slugs [32,33]. Caffeine has three methyl groups in a purine ring and is commonly named as 1, 3, 7 tri-methyl xanthine. Caffeine is known to increase intracellular Ca^++ ^by promoting the discharge of Ca^++ ^sequestered in mitochondria and smooth endoplasmic reticulum [34,35] which in turn is known to block pinocytosis in slime molds [35]. Adenosine is a non-competitive inhibitor of cAMP receptors [36-38] and caffeine reversibly inhibits the cAMP-dependent activation of the adenylate cyclase [34,39].

Here, we investigated the effect of adenosine and caffeine on the aggregate size of several Dictyostelia species across all 4 slime mold groups. We present evidence that these compounds change the aggregate size by modulating cell number and size, countin expression, cytosolic glucose levels, cell movement, and cell-cell adhesion.

## Methods

### Cell culture

All wild type strains of *Dictyostelium *were cultured on SM/5 agar plates in association with *K. aerogenes *at room temperature (22°C) except AX2 which was grown in HL5 media. The *Dictyostelium *mutant strains were grown in axenic HL5 medium (28.6 g bacteriological peptone (Oxoid), 15.3 g yeast extract (Oxoid), 18 g Maltose (Sigma), 0.641 g Na_2_HPO4 (Merck) and 0.49 g KH_2_PO_4 _(Fluka) per litre, pH-6.4) containing antibiotics (200 units/ml penicillin and 200 μg/ml streptomycin sulphate) at 22°C with constant shaking (180 RPM). *Polysphondylium pallidum *PN500 (a kind gift from Dr. Edward Cox, Princeton University) was grown on GYP agar plates (1 g glucose, 2 g bacteriological peptone (Oxoid), 0.2 g yeast extract (Oxoid), 4.2 g KH_2_PO_4 _(Fluka) 2.7 g Na_2_HPO_4 _(Merck) and 15 g agar per litre, pH-6.4) in association with *E. coli *B/r*^- ^*at 22°C with 70% relative humidity. When there was visible clearing of the bacterial lawns, the cells were harvested by washing the plates with ice-cold KK2 buffer (2.25 g KH_2_PO_4 _and 0.67 g K_2_HPO_4 _per litre H_2_O, pH 6.4). Thereafter, the amoebae were plated at a density of 1 × 10^6 ^cells/cm^2 ^on non-nutrient agar plates (KK2 buffer containing 15 g agar per litre, pH 6.4) containing the indicated concentration of adenosine or caffeine and we scored for changes in the aggregation pattern under a microscope (Nikon SMZ1000 and Nikon eclipse 80i).

### Cell division assay

The cell division kinetics of AX2 cells was performed in three different conditions: 1. In the presence of adenosine or caffeine: We inoculated 2 × 10^6 ^in test tubes having 10 ml of HL5 medium with either adenosine or caffeine (2 mM) and incubated at 22°C with constant shaking (180 RPM). The kinetics was monitored by counting the number of cells with a haemocytometer at regular intervals under a light microscope. 2. Growth kinetics of starved cells in the presence of adenosine/caffeine: We harvested vegetative cells grown in HL5 media (without caffeine/adenosine) and washed twice with ice cold KK2 buffer. We inoculated 1.2 ± 0.12 × 10^6 ^cells in 10 ml of Sorensen buffer (2 g KH_2_PO_4 _and 0.29 g Na_2_HPO_4 _per litre H_2_O, pH 6.4) containing (3 mM) caffeine/adenosine/10 mM glucose or combinations of these compounds with 10 mM glucose. After 9 hours of incubation at 22°C, we counted the number of cells with a haemocytometer. 3. Growth kinetics during early developmental stages: Cells that were not exposed to the drugs earlier during growth were allowed to develop with caffeine or adenosine and subsequent to aggregate formation, they were dissociated and the cell number was counted. Aggregates were allowed to form in 90 mm Petri dish submerged in Sorensen phosphate buffer containing either caffeine or adenosine (3 mM). The aggregates were dissociated at the indicated time points by incubating them with dissociation buffer (50 mM Tris-HCl (pH-7.5), 5 mM EDTA, 0.2% Pronase-E) and numbers of cells were counted in a haemocytometer.

### Cell size and cell volume measurements

To measure cell size of starving cells, Sorensen buffer and Sorensen buffer + 120 mM sorbitol containing the indicated concentrations of caffeine and adenosine were used. Sorensen buffer was complemented with sorbitol to maintain the osmolarity [40] in case caffeine or adenosine perturbs a change in the cell size. 5 × 10^6 ^cells/ml were incubated in with constant shaking at 150 RPM on a horizontal shaker for 6 hours. To measure the size of vegetative AX2 cells, we replenished the medium with HL5c (Formedium-HL5 medium with glucose) medium containing 5 mM adenosine or 5 mM caffeine when cells reached a 70% confluence. After 6 hours of incubation, we collected the cells and measured the cell sizes using Casy^R ^TT cell counter machine. The cell volume was measured by the scale given in packed cell volume tubes (PCV).

### Cell movement assay

AX2 cells were plated in non-nutrient agar containing either 2 mM adenosine or 2 mM caffeine and individual cell movement was recorded for 8 hours after starvation [26]. For each cell, the field of view was recorded for 5 minutes and cell movement was then calculated in 1 μm/min intervals [26]. Bulent screen recorder software was used to record the movement of cells and the entire observation was carried out under a Nikon eclipse 80i upright microscope.

### Cell adhesion assay

2 × 10^7 ^cells were suspended in 10 ml of KK2 buffer containing 2 mM adenosine or 2 mM caffeine and incubated at 22°C with constant agitation (180 RPM). Cell-to-cell adhesion assay was carried out by scoring for the presence of solitary cells or clumps of cells with two or more cells adhered to each other [19,41].

### Glucose assay

Axenically grown AX2 cells were harvested and re-suspended in HL-5 medium or in Sorensen buffer (starvation) at a density of 8 × 10^6 ^cells/ml and incubated for 6 hours at 22°C with constant shaking in the presence of 3 mM adenosine or 3 mM caffeine. The amoebae were harvested at 1200 RPM for 10 minutes at 4°C in PBM buffer (20 mM KH_2_PO_4_, 10 μM CaCl_2_, 1 mM MgCl_2_, pH 6.1; [10] and were lysed by freezing them at -80°C for 8 hours. 35 μl from the supernatant was mixed with 200 μl of glucose assay reagent (GAHK20; Sigma-Aldrich, USA) in a 96 well microtiter plate, incubated for 15 minutes and the absorbance was measured at 340 nm. From a similar sample, 2.0 μl of supernatant was mixed with 250 μl of Bio-Rad protein assay reagent, incubated for 20 minutes and absorbance was measured at 595 nm for protein estimation.

### Western blot analysis

For immuno-blotting of Cad-1 and CsaA cell adhesion proteins, extracellular cAMP phosphodiesterase (PdsA) and Countin, we grew AX2 cells in HL5 media and starved in Sorensen buffer at a density of 1 × 10^7 ^cells/ml in the presence or absence of the drug at 22°C with continuous shaking (150RPM). 5 × 10^6 ^cells were lysed in 200 μl cell lysis buffer (2% SDS, 0.5 M Tris- pH-6.8) containing 1% mercaptoethanol and the mixture was heated at 95°C for 5 min [20]. 20 μl of the cell lysate was electrophoresed either in 13% (Countin and PdsA) or in 10% (Cad-1, CsaA,) polyacrylamide gels. Equal loading of the protein lysates was checked by staining the nitro-cellulose membrane with ponceau-S dye after blotting. The anti-Dd Cad-1 (1:8000) polyclonal antibody, anti-PdsA (1:1000) polyclonal antibody, anti-CsaA (1:10 from the mice supernatant) monoclonal, anti-countin (1:500), polyclonal antibody were incubated over night at 4°C. Subsequently, secondary HRP conjugated antibodies was incubated for one hour at room temperature along with membrane.

### Cell mixing experiments

For reconstituting *acaA^- ^*cells with AX2 cells, we grew both the strains in HL5 medium separately. After pelleting, the cells were washed twice with ice cold KK2 buffer, counted and mixed in two different ratios (4:1 and 2:3). Subsequently, the mixed cells were (1 × 10^7 ^cells/ml) starved together in Sorensen buffer for 5 hours at the density of the 1 × 10^7 ^cells/ml and plated at density of 1 × 10^6 ^cells/cm^2 ^on non-nutrient plates containing either adenosine or caffeine.

### Statistical analysis

The statistical analyses were carried out using the Microsoft Office Excel 2003 software. The statistical significance of experiments was confirmed by performing either One-way ANOVA (Analysis of Variance) or Student's t-test (paired).

## Results

### The effect of adenosine and caffeine on aggregate size is conserved in slime molds representing four evolutionary groups

**Figure 1:**
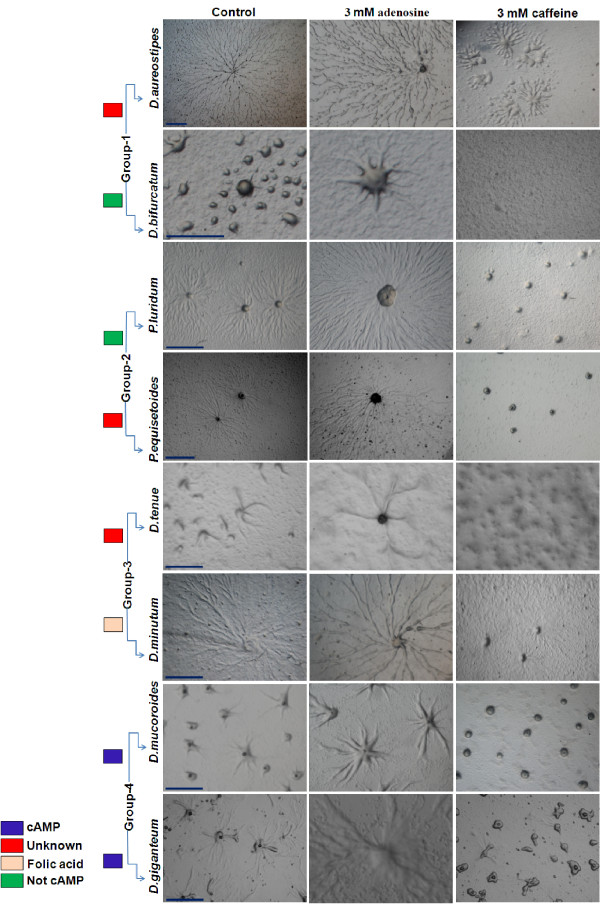
**Effect of adenosine and caffeine on the aggregate size of different species of *Dictyostelium *and *Polysphondylium***. In all the species examined, large aggregates were formed in the presence of adenosine and contrastingly, with caffeine many small aggregates were formed. The aggregates were developed as described in material and methods. Scale bar = 1000 μm.

To determine if there is an evolutionarily conserved action of adenosine and caffeine, we examined their effect on aggregate size in eight other slime mold species representing four lineages [8]. In all the species studied, the aggregates were larger in the presence of adenosine and were smaller in the presence of caffeine (Figure 1). In group 4 species (*D. giganteum *and *D. mucoroides*), many small aggregates and few large aggregates, were formed in the presence of caffeine and adenosine, respectively. Group 3 species (*D. minutum *and *D. tenue*) displayed a similar phenotype to that of group 4 species with caffeine and adenosine (Figure 1). Further, we monitored the aggregation pattern of group 2 (*Polysphondylium luridum *and *P. equisetoides*) and group 1 (*D. bifurcatum *and *D. aureostipes*) species and observed similar phenotypes to group 4 and 3 species. This data strongly suggests that in spite of using structurally unrelated chemotactic messengers, the aggregation process seems to be conserved in all cellular slime molds and the mechanisms regulating aggregate size may also be common.

**Figure 2:**
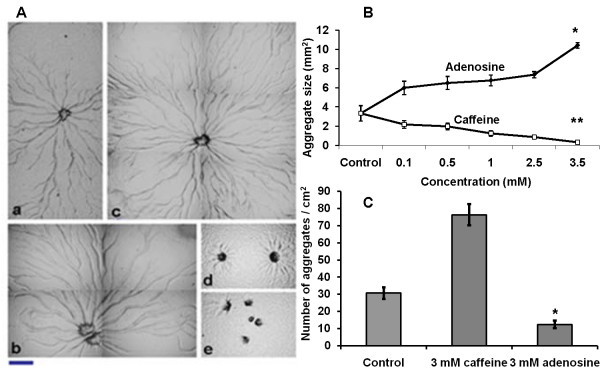
**Effect of adenosine and caffeine on aggregation pattern of *Dictyostelium *and *Polysphondylium***. A) Aggregation pattern of the *Polysphondylium *cells a) Control (b) with 0.5 mM (c) with 1 mM adenosine. Small aggregate formation in the presence of caffeine (d) 1.0 mM (e) 2.5 mM. Scale bar = 200 μm. *Polysphondylium *amobae were plated at a density of 1 × 10^6 ^cells/cm^2 ^on KK2 plates containing caffeine or adenosine and were allowed to form aggregates in the dark and humid conditions. (B) Aggregate size (Student's t-test, *p < 0.001, **p < 0.004). (C) The number of aggregates (Student's t-test, *p < 0.001). There were few aggregation centres, in the presence of adenosine. Many, small aggregates were formed in the presence of caffeine. Numbers of aggregates were counted in 1 cm^2 ^area. All values represent the mean ± standard error (n = 10).

Further, we chose *P. pallidum *that makes use of a modified dipeptide glorin [6] as a chemoattractant to quantify the extent to which adenosine and caffeine could exert their effect on species known to use structurally unrelated chemoattractants, As in *Dictyostelium*, there is a direct correlation between aggregate size and the concentration of the compounds tested (Figure 2A, B, Additional file 1, Fig S1). The aggregate and slug size increased in the presence of adenosine (Figure 2B, Additional file 1, Fig S1) and at 3.5 mM adenosine, the average size of the aggregates were 10.4 ± 0.50 mm^2 ^(p < 0.004) while in controls (without compounds), it was 3.33 ± 0.9 (mm^2^). Aggregates and slug formed in the presence of caffeine were many and small (Figure 2A, Additional file 1, Fig S1), an effect mimicked by having adenosine deaminase (50 units per ml) in the medium (Additional file 2, Fig S2). Adenosine deaminase is known to convert intracellular adenosine into inosine and therefore the concentration of adenosine will go down when cells are treated with adenosine deaminase [42]. At 3.5 mM caffeine, the average size of aggregates was 0.32 ± 0.07 mm^2 ^(p < 0.001). Since the aggregate size was large in the presence of adenosine, only few aggregates were formed in a defined area though the initial cell density was the same in all plates. With caffeine, numerous small aggregates were formed and also many cells stayed solitary without participating in aggregate formation (Figure 2C). In the presence of 3 mM adenosine, the average number of aggregates per cm^2 ^was 12.4 ± 2.3 and with 3 mM caffeine it was 76.2 ± 6.2 with (p < 0.001). The number of aggregates in controls (without the compounds) was 30.6 ± 3.4. The aggregate size was measured as described by Tang and Gomer 2008 [43] with slight modification. During development, aggregate territories become compact in a particular area and the number of aggregates remains unchanged prior to stream breaking. So we estimated the size of aggregates by obtaining the number of aggregates in an area of 100 mm square. The aggregate size was ascertained by the total number of aggregates per unit area. This study suggests that adenosine favours large aggregation territories and caffeine does exactly the opposite effect in forming small territories and both these compounds could influence cell proliferation rates and cell size, the major determinants of aggregate size.

Since the effects of adenosine and caffeine were similar in all *Dictyostelium *and *Polysphondylium *sp. examined, the mechanisms that regulate aggregate size may also be common. We hence chose *D. discoideum *to know the factors that might contribute to changes in aggregate size, pattern and streaming in the presence of adenosine or caffeine.

### Adenosine accelerates cell-division rates while caffeine slows it down

**Figure 3:**
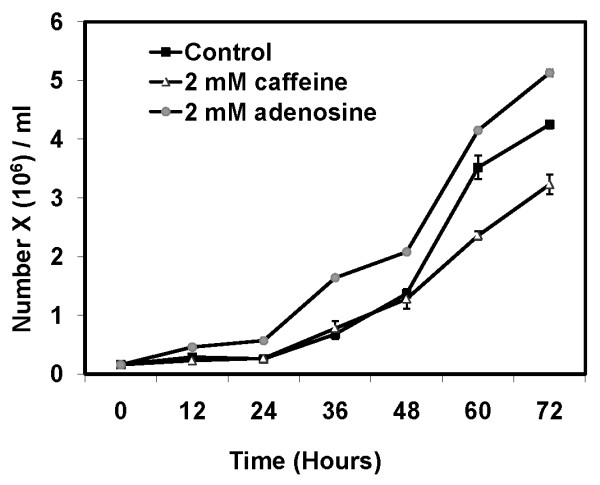
**Cell division kinetics of AX2 cells in presence of 2 mM caffeine or 2 mM adenosine**: 2 × 10^5 ^cells/ml were inoculated in HL 5 medium containing either adenosine or caffeine with constant shaking at 180 RPM and cell division was estimated by counting the number of cells with a haemocytometer at regular intervals. The values represent mean ± standard deviation (n = 4) (Student's t-test, *p < 0.01, **p < 0.001).

The total cell number and the individual cell size in an organism determine its size [12,13]. If adenosine or caffeine changes the cell number/size or both that would result in altered aggregate sizes. The kinetics of cell division, cell size and the volume of AX2 cells in growing, starving and developing conditions in the presence of each compound were measured. The cell division rates were monitored in HL5 medium every eight hours for a total duration of 72 hours. Adenosine significantly increased the division rates while caffeine retards the division rates (Figure 3). In the presence or absence of 2 mM adenosine or caffeine, the cell density reached 4.2 ± 0.13 × 10^6 ^cells/ml, 5.12 ± 0.10 × 10^6 ^cells/ml (p < 0.01), and 3.2 ± 0.03 × 10^6 ^cells/ml (p < 0.001), respectively (Figure 3).

We further examined the division rates upon caffeine or adenosine exposure, during development. For this, we seeded 3.0 ± 0.5 × 10^6 ^cells in 90 mm Petri dishes submerged with Sorensen buffer (SB) in the presence or absence of adenosine or caffeine. After 9 hours, we dissociated the aggregates and found 3.83 ± 0.14 × 10^6 ^cells in controls (Table 1). Plates containing adenosine and caffeine had 4.33 ± 0.51 × 10^6 ^and 3.27 ± 0.43 × 10^6 ^cells, respectively (p < 0.05) (Table 1). This result suggests that additional cell divisions taking place during the early developmental phase, significantly contributing to an increased or decreased cell number in the aggregates in the presence of adenosine and caffeine, respectively.

**Table 1:** Cell division during development in the presence of either caffeine or adenosine

Zero hours	3.0 ± 0.5 × 10^6^
**After 9 hours**	
**Control**	3.83 ± 0.14 × 10^6^
**3 mM adenosine**	4.33 ± 0.51 × 10^6 ^
**3 mM caffeine**	3.27 ± 0.43 × 10^6 ^

Number of cells in aggregates (Student's t-test, p < 0.05, n = 3)

Glucose is a key nutrient regulating cell growth and viability by activating the TOR complex I (mTOR in mammals; [14,44]). To check if adenosine mimics the action of glucose in enhancing the cell growth, we counted the total cell number during starvation (in Sorensen buffer) and this number was compared to the number of cells that were exposed to the compounds (adenosine/caffeine/glucose) during starvation. In controls, 1.2 ± 0.12 × 10^6 ^cells/ml was inoculated and after 9 hours of starvation, the cell number increased to 1.43 ± 0.11 × 10^6 ^cells/ml (Table 2). In the presence of adenosine, caffeine and glucose the cell numbers were 1.58 ± 0.2 × 10^6^, 1.23 ± 0.75 × 10^6 ^and 2.25 ± 0.39 × 10^6 ^cells/ml, respectively (Table 2). The cell number was even higher in samples containing both caffeine/adenosine and glucose. The numbers of cells in the presence of caffeine + glucose were 1.9 ± 0.30 × 10^6 ^cells/ml whereas with adenosine + glucose it was 2.59 + 0.33 × 10^6 ^cells/ml (Table 2). Taken together, these results imply that with adenosine by an unknown mechanism induce excessive cell proliferation contributing to a large number of cells available for aggregation. Supplementing caffeine in the medium decreased the total cell number and hence many small sized aggregates were formed. Besides, a number of cells remained solitary without aggregating and this could also be another factor responsible for small aggregate formation in the presence of caffeine.

**Table 2:** Number of cells in starvation buffer (One-way ANOVA, p < 0.001, n = 5, it was performed to check if the values obtained in different treatments were significantly different from the control [without compounds]).

Zero hours	1.2 ± 0.12 × 10^6^
**After 9 hours**	
**Control**	1.43 ± 0.11 × 10^6 ^
**3 mM adenosine**	1.58 ± 0.20 × 10^6 ^
**3 mM caffeine**	1.23 ± 0.75 × 10^6^
**10 mM glucose**	2.25 ± 0.39 × 10^6^
**3 mM adenosine + 10 mM glucose**	2.59 ± 0.33 × 10^6 ^
**3 mM caffeine + 10 mM glucose**	1.90 ± 0.33 X10^6^

Because the aggregate size might also be influenced by the size of individual cells that make up the colony, we measured the cell diameter and volume of vegetative and starved AX2 cells treated with either caffeine or adenosine. It is reported that 120 mM Sorbitol shrinks the amoebae size by 30% [45]. To ensure that caffeine and adenosine do not cause an osmotic imbalance thereby affecting the cell size, we independently starved the amoebae in an SB buffer containing 120 mM sorbitol. Caffeine treated cells were smaller and adenosine supplemented medium had larger cells compared to controls (Table 3). The cell diameter was measured after 6 hours for both vegetative and starved cells in all the conditions mentioned. The average cell diameter of the adenosine-treated vegetative cells was 9.93 ± 0.03 μm, whereas caffeine-treated cells had a diameter of 9.30 ± 0.13 μm. The diameter of the amoebae from controls was 9.75 ± 0.24 μm. The diameter of cells starved (control) in SB and SB + sorbitol were 7.83 ± 0.10 μm and 8.87 ± 0.05 μm, respectively. In the presence of adenosine in SB and SB + sorbitol, the average cell diameter were 8.20 ± 0.12 μm and 9.25 ± 0.05 μm, respectively, whereas with caffeine cell diameter were 6.59 ± 0.19 μm and 8.50 ± 0.30 μm (Table 3).

**Table 3:** Effect of caffeine and adenosine on cell size.

AX2	Sorensen's buffer	Sorensen's buffer +120 mM sorbitol	Hl5c
**Cell volume**			
**Control**	7.83 ± 0.10	8.87 ± 0.05	9.75 ± 0.24
**5 mM adenosine**	8.20 ± 0.12 (p < 0.05)	9.25 ± 0.05 (p < 0.003)	9.93 ± 0.03 (p < 0.2)
**5 mM caffeine**	6.59 ± 0.19 (p < 0.02)	8.39 ± 0.05 (p < 0.004)	9.30 ± 0.13 (p < 0.05)

All values represent the mean ± standard deviation of 3 independent experiments. Student's t-test was performed to check the significance of the experiments.

In the presence of caffeine, the volume of starved cells decreased by 17.1% and in the presence of sorbitol and caffeine, the volume reduced by 14.3%, a direct measure of the cell size (Table 4). However, there was a marginal increase in cell volume (2%) in the presence of adenosine. The small increase in the volume and enlarged size of the amoebae in the presence of adenosine may be another reason why the aggregates may be larger than the controls. Because of a significant reduction in cell size in the presence of caffeine, the aggregates were also smaller. This data suggests that cell size and volume could be significant factors contributing to altered aggregate sizes in the presence of these compounds.

**Table 4:** Effect of caffeine and adenosine on cell volume

AX2	Sorensen's buffer	Sorensen's buffer +120 mM sorbitol
**Cell size**		
**Control**	100%	100%
**5 mM adenosine**	102%	100%
**5 mM caffeine**	82.9%	85.7%

### Both adenosine and caffeine enhance cell-cell adhesion

**Figure 4:**
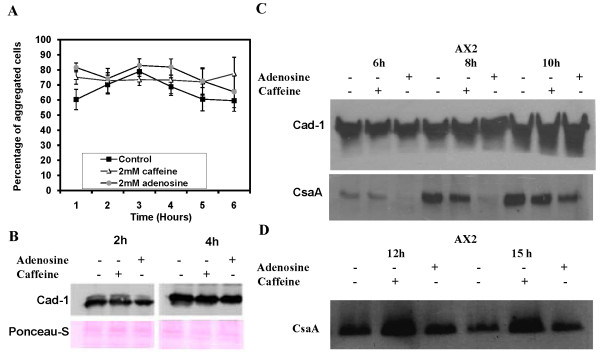
**Cell adhesion in *Dictyostelium *amobae**. A) Cell-cell adhesion in the presence of 2 mM adenosine or 2 mM caffeine. It was carried out by scoring for the presence of single cells or clumps of cells with two or more cells adhered to each other. All values represent the mean ± standard error of 7 independent experiments. B) Expression pattern of Cad-1 proteins in AX2 cells. The Cad-1 expression level was checked in cells developed for 2 h and 4 h in Sorensen buffer in the presence or absence of either caffeine or adenosine. C) Expression levels of Cad-1 and CsaA in AX2 cells developed for 6 h, 8 h, 10 h, 12 h and 15 h in the presence or absence of either caffeine or adenosine.

Cohesive binding among cells determines the size and pattern of aggregation [19] and this factor could account for increased size and compactness of aggregation streams formed when cells were developed in presence of adenosine and caffeine, respectively. As monitored by cell-adhesion assays both adenosine and caffeine enhanced cell cell-to-cell adhesion with values of 65.39 ± 10.48% and 77.46 ± 10.89%, respectively (Figure 4A). For the control, the value is 59.55 ± 7.04% after 6 hours of starvation.

If both adenosine and caffeine enhance cell adhesion, then one would expect caffeine to induce large aggregate formation which never happened. To gain molecular insights of this differential response with caffeine, western blot analysis was carried out to examine if caffeine and adenosine affect the expression of the cell-cell adhesion glycoproteins Cad-1 and CsaA. During early aggregation stages, Cad-1 expression was not significant in the presence of adenosine or caffeine compared to untreated controls. A reduced Cad-1 expression by 20% and 25% was observed at 2 h and 4 h of development, respectively (Figure 4B). At 6 h of development, Cad-1 expression was almost equal with both caffeine and adenosine and further increased two-fold at 8 h and 10 h of development.

At 6 hours of starvation, i.e. at the late stage of aggregation, the expression of the developmentally regulated cell adhesion protein CsaA could be observed. Since the aggregate formation was delayed by 4 hours in the presence of both these compounds (Additional file 3, Additional file 4, Fig S3), the expression level of CsaA was also not seen. With adenosine, CsaA expression was delayed until after 8 h of development and increased its expression at 12 h and 15 h of development (Figure 4C). With caffeine, CsaA expression got reduced two-fold at 6 h and 8 h of development but increased its expression two- to three-fold higher at 10 h, 12 h, and 15 h of development (Figure 4C). Delayed expression of the cell adhesion proteins in the presence of both the compounds correlates with the development timing of the cells. At late aggregation stages, the cell adhesion proteins (CsaA and Cad-1) are strongly expressed in the presence of adenosine or caffeine. The elevated expression of these proteins might favour strong cell-cell adhesion thereby favouring large aggregate formation in the presence of adenosine. In spite of an increase of the cell adhesion protein expression with caffeine, the aggregates formed were small and it is likely that caffeine may be involved in maintaining the integrity of small but streamless aggregates. Though caffeine favoured small aggregate formation, still there were many aggregation centers each compact on its own and the expression of cell adhesion proteins may be responsible for this small but compact aggregate formation. These data suggest that enhanced expression of cell adhesion proteins results in increased binding of cells with each other resulting in large aggregate formation in the presence of adenosine.

### Caffeine and adenosine alter the aggregate size by affecting the cytosolic glucose levels

**Figure 5:**
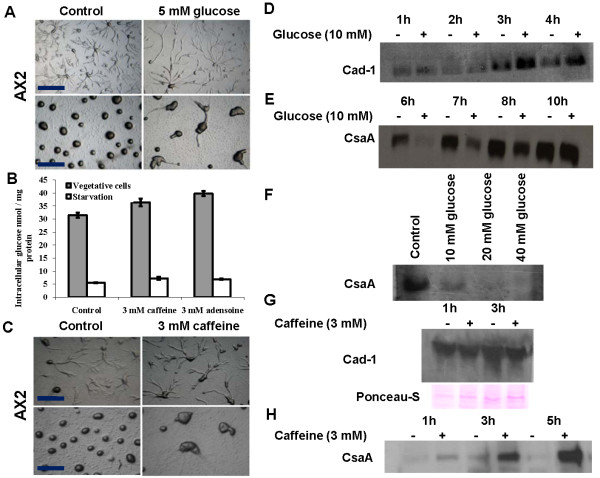
**Cells grown in the presence of caffeine led to an increase in the levels of intracellular glucose or developing cells with extracellular glucose led to altered aggregate size and cellular adhesion protein Cad-1 and CsaA expressions**: A) Effect of extracellular glucose on aggregate size. B) Cytosolic glucose levels of vegetative and developing cells in the presence or absence of either caffeine or adenosine. The glucose level was estimated as described in materials and methods. The values represent mean ± standard deviation (n = 8), *P < 0.1, **P < 0.01 (Student's t-test). C) Aggregates formed from cells grown in the HL5 medium in the presence or absence of caffeine for the 24 hours and subsequently harvested, washed and developed in the non nutrient agar in the absence of caffeine. D) Cad-1 expression levels in AX2 cells developed for 1 h, 2 h, 3 h and 4 h in Sorensen buffer in the presence or absence of 10 mM glucose. E) CsaA expression levels in cells developed for 6 h, 7 h, 8 h and 10 h in Sorensen buffer in the presence or absence of 10 mM glucose. F) CsaA expression levels in cells developed for 6 h in Sorensen buffer in the presence of different concentrations of glucose. G) Expression pattern of Cad-1 proteins in AX2 cells. Cells were grown in the presence or absence of 3 mM caffeine for 24 hours and Cad-1 expression levels were checked in cells developed for 1 h and 3 h in Sorensen buffer in the absence of caffeine. H) CsaA levels in AX2 cells grown in the presence or absence of caffeine. CsaA levels were checked in the cells developed for 1 h, 3 h and 5 h in Sorensen buffer in the absence of caffeine.

The effect of caffeine and adenosine on cell size and cell growth can be mediated through changes in the steady-state cytosolic glucose concentrations. The cytosolic glucose concentration can be a major factor determining cell shape, cell size, cell movement and cell-cell adhesion, consequently contributing to changes in aggregate size [10]. Indeed aggregate size can be altered during development by supplementing glucose during growth or development [10]. When AX2 cells were allowed to develop in non-nutrient medium containing 5 mM glucose, it resulted in the formation of large aggregates (Figure 5A) though there was a 1 hour delay in the formation of the aggregation centres. To confirm if altered aggregate sizes observed in the presence of caffeine or adenosine are due to changes in the cytosolic glucose levels, its concentration was monitored in vegetative and starved cells. Cytosolic glucose levels increased in both vegetative and starved cells (6 hours) that were either grown or starved in the presence of each compound (Figure 5B). With 3 mM adenosine, cytosolic glucose levels of vegetative and starved cells were 39.71 ± 1.00 and 6.9 ± 0.22 nmol/mg protein, respectively. With 3 mM caffeine, the glucose levels were 36.36 ± 1.47 and 7.2 ± 0.56 nmol/mg protein, respectively. The values observed were significantly higher (p < 0.01) than in untreated controls, which was 31.44 ± 0.96 nmol/mg protein (vegetative cells) and 5.5 ± 0.10 nmol/mg protein (starved cells) (Figure 5B). Caffeine induced increase in cytosolic glucose levels could be mimicked by supplementing exogenous glucose during starvation. Cells starved with 5 mM glucose had higher glucose levels than untreated controls. With 5 mM glucose, the cytosolic glucose level was 9.82 ± 2.73(p < 0.001) nmol/mg proteins. In untreated control (absence of glucose) the cytosolic glucose level was 4.77 ± 1.35 nmol/mg proteins (Additional file 5, Fig S4).

Glucose levels are known to affect the colony size and we monitored the size of aggregates formed from cells that were grown in the presence of 3 mM these compounds. There was a precocious development (by 2 hours) when amoebae were grown in the presence of 3 mM caffeine. Vegetative cells treated with caffeine and developed in its absence formed large aggregates (Figure 5C) and the size was even larger from those cells that were exposed to adenosine during vegetative growth (not shown). This suggests that the regulation of aggregate size by either caffeine or adenosine acts via the glucose sensing pathway. The caffeine effect appeared to be stage specific and if the treatment were in vegetative stage followed by no caffeine during development, then large aggregates were formed. However, if the cells were grown in the absence of caffeine and developed in its presence later, then small aggregates were formed. An increase in intracellular glucose levels induced by caffeine during growth may be directly responsible for large aggregate formation.

To test whether increasing cytosolic glucose induces an increased expression of cell adhesion protein, the levels of Cad-1 and CsaA and were quantitated by western blot of cells grown with caffeine and developed in the presence or absence of glucose (without caffeine). The delayed development in the presence of 5 mM glucose can also be monitored by checking the expression levels of CsaA, which is a development regulatory protein [46]. In the presence of glucose, there was an increase in Cad-1 expression and a decrease in CsaA expression compared to controls without glucose. With 10 mM glucose, Cad-1 expression increased by 1.5 to 2.5 fold at 1 h, 2 h, 3 h and 4 h of development (Figure 5D). CsaA expression gets repressed in the presence of glucose with barely detectable expression at 6 h of development. After 10 h of development in the presence of glucose, CsaA expression levels were comparable to the untreated controls (Figure 5E). Irrespective of the glucose concentrations tested (10 mM, 20 mM and 40 mM) CsaA expression was abolished at 6 h of development (Figure 5F).

In contrast, the Cad-1 levels from cells grown in the presence of 3 mM caffeine and developed in its absence were decreased by 25% and 50% at 1 h and 3 h of development, respectively (Figure 5G). CsaA expression was seen during early aggregation stages and increased gradually at 1 h, 3 h and 5 h of development (Figure 5H). Although vegetative cells exposed to caffeine mimics the effect of glucose (aggregate size), the developmental timing and cell adhesion protein expressions were different in the presence of these compounds. A reduced Cad-1 expression level in the presence of 3 mM caffeine (which is known to increase the cytosolic glucose levels) and increased expression of CsaA in the presence of extracellular glucose suggests that there is compensatory effect of these cell adhesion proteins. If the expression of one cell adhesion protein is repressed, the other cell adhesion protein expression gets stronger implying that they complement each other in increasing the overall cell adhesion.

**Figure 6:**
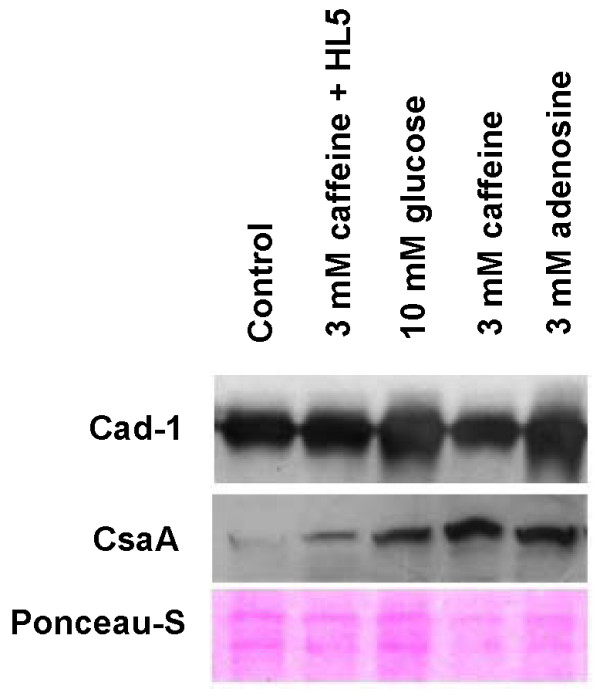
**Expression levels of cell adhesion proteins Cad-1 and CsaA during aggregation: The aggregates were developed on non-nutrient agar plates in the presence or absence of caffeine, adenosine or 10 mM glucose**. The fully streamed aggregates were washed off with ice chilled Sorenson's buffer and subsequently cells were treated with lysis buffer. 5 μg of the protein was loaded in each well and the equal loading of checked by staining the membrane with Ponceau-S dye. 2^nd ^lane of the blot represents the expression of Cad-1 and CsaA form the aggregate developed in the absence of compounds of the AX2 cells grown in the presence of 3 mM caffeine. The expression levels of these proteins were quantified using image J software (NIH-USA) and the changes in expressions were normalized with loading control.

The aggregate formation was delayed in the presence of adenosine or caffeine and correspondingly the CsaA expression levels also gets altered indicating the developmental status of the cells contributes to the changed aggregation pattern also. We performed western blot of Cad-1 and CsaA in early aggregation stages. Interestingly, there was enhanced Cad-1 and CsaA expression with these compounds (Figure 6). There was a two fold increase in Cad-1 expression from aggregates developed in the presence of either 10 mM glucose or 3 mM adenosine. However, in the presence of caffeine there was no significant increase of Cad-1 expression from the aggregates or from cells grown with caffeine and developed in absence. The CsaA expression in the presence of 10 mM glucose, 3 mM caffeine and 3 mM adenosine increased by 2 fold, 4 fold and 4 fold respectively (Figure 6). CsaA expression from cells grown in the presence of caffeine and developed in its absence also increased by 2 fold (Figure 6). Taken together, these studies strongly suggest that increasing cytosolic glucose levels either by direct or indirect means indeed increased the expression of cell adhesion proteins Cad-1 and CsaA. The aggregates formed with either glucose or with adenosine had higher Cad-1 and CsaA expression which resulted in large aggregate formation. However, the integrity of the aggregate formation with caffeine treatment may be due to CsaA expression alone and not Cad-1.

### Caffeine and adenosine alter strength of cAMP relay

If the cAMP relay is perturbed, it affects aggregate size [43]. Chemotaxis, cell motility, and strength of cAMP relay during aggregation are regulated by the adenylyl cyclase AcaA, the cAMP phosphodiesterases Pde4 and PdsA, and the cAMP phosphodiesterase inhibitor PDI [21-24]. Pde4, a membrane bound extracellular cAMP phosphodiesterase regulates cAMP concentration during development [21]. The extracellular cAMP phosphodiesterase (PdsA) is known to regulate group migration during aggregation by degrading the secreted chemoattractant. cAMP phosphodiesterase (*pdsA*^-^) mutants have an impaired relay and adenylyl cyclase (*acaA^-^*) null cells don't secrete cAMP and both mutants do not chemotax and stream [22,29]. The cAMP phosphodiesterase inhibitor (PDI) is a key component of the cAMP relay mechanism that helps in regulating the aggregate size. *pde4*^- ^cells do not stream and forms small clumps of cells. The *pdiA^- ^*cells rarely form spirals and aggregates are small [23].

**Figure 7:**
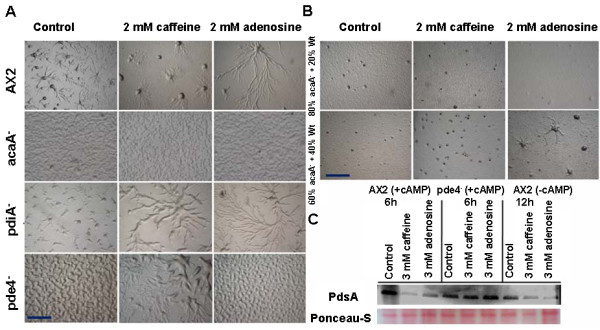
**Caffeine alters cAMP signal relay by affecting cAMP phosphodiesterase synthesis (PdsA)**. A) Aggregation pattern of AX2, *acaA^- ^, pdiA^- ^*and *pde4^- ^*cells in the presence or absence of either caffeine or adenosine. Caffeine rescued the *pdiA*^- ^and *pde4^- ^*cells into wild type pheonotype. B) Rescuing of *acaA^- ^*null phenotype by mixing the AX2 cells with *acaA^- ^*cells in 4: 1 and 3: 2 ratio and checking the aggregation in presence of either caffeine or adenosine. C) Western blot of PdsA protein, to check the expression levels in vegetative AX2 cells pulsed or unpulsed with cAMP and *pde4^- ^*cells pulsed with cAMP.

If caffeine or adenosine alters the strength of the cAMP relay, then the streaming and the size of aggregates of these mutants might be rescued. To test this, we monitored the aggregation pattern of AX2, *pdiA*^-^, *pde4*^-^ and *acaA*^-^ null cells in the presence of either adenosine or caffeine. The *acaA*^-^ cells are aggregation defective and neither adenosine nor caffeine was able to rescue this phenotype. In contrast, *pdiA*^- ^cells formed very small aggregates and both caffeine and adenosine rescued the phenotype of *pdiA*^- ^and induced the formation of larger aggregate (Figure 7A). Aggregates of *pdiA*^- ^formed in the presence of caffeine are comparatively larger than the aggregates formed with adenosine (Figure 7A). Finally, we investigated the effect of these drugs on the aggregation pattern of *pde4*^- ^cells that do not stream and form only small clumps of cells. In the presence of caffeine, *pde4*^- ^cells streamed and formed large aggregates while adenosine showed no effect (Figure 7A). This data suggests that caffeine by an unknown mechanism acting on other proteins that could possibly taking over the function of PDI and Pde4 and this may possible by activating other phosphodiesterases thereby altering the strength of the cAMP relay signal. Adenosine partially rescues the function of PDI but its effect is not as potent as caffeine and it has no effect on Pde4.

a*ca- *cells have impaired cAMP synthesis and remain single celled. However, if *aca- *cells are pulsed and developed in the presence of exogenous cAMP, aggregates phenotype can be rescued. If *aca- *cells were reconstituted with AX2, the secreted cAMP from AX2 might rescue *aca- *cells there by aggregating together. To test whether caffeine and/or adenosine affect the levels of cAMP secreted, we mixed a large fraction of *aca- *cells impaired in cAMP synthesis with AX2 in a ratio of 4:1 (*aca^- ^*: AX2) and 3:2 (*aca^- ^*: AX2). If there is sufficient cAMP production, this might rescue the aggregation phenotype of *aca- *cells and if either caffeine or adenosine affects this process, that could be assayed by the presence of aggregate formation. By scoring the aggregate number and size in reconstituted experiments, we could ascertain if there was a change in cAMP levels in presence of adenosine/caffeine. Mixing *acaA*^- ^cells with AX2 cells in a 3:2 (*aca^- ^*: AX2) ratio rescued aggregation phenotype and in 4:1 ratio, aggregation centres alone were formed with no spirals (Figure 7B). In *aca^-^*and AX2 combinations, the number of small aggregation centers formed in the presence of caffeine was higher in 3:2(*aca^- ^*: AX2) ratios than 4:1 (*aca^- ^*: AX2) ratio (Figure 7B). Aggregates formed in the presence of adenosine were larger than the controls in 3:2 (*aca^- ^*: AX2) ratios (Figure 7B). Based on these experiments, it is likely that both caffeine and adenosine prevent cAMP degradation possibly by affecting pdsA levels and rescue the *acaA*- phenotype in the presence of wild type cells.

Pde4 regulates cAMP concentration during development [21]. Caffeine might rescue *pde4*- function by inducing other phosphodiesrerases such as PdsA. To estimate indirectly the level of extracellular cAMP degradation in the presence of caffeine and adenosine, we performed western blots of PdsA expression in AX2 (pulsed with 30 nM cAMP for 5 hours at 6 minutes intervals), *pde4*^- ^cells (pulsed with cAMP) and AX2 unpulsed. In AX2 cells pulsed with cAMP, *PdsA *expression at 6 hours of development was higher than from unpulsed AX2 cells developed for 12 hours (Figure 7C). In the presence of 3 mM adenosine or caffeine, PdsA levels were reduced in both pulsed and unpulsed AX2 cells. In *pde4*^- ^cells, the PdsA levels at 6 h of development, in the presence of caffeine and adenosine increased by 25% and 40% respectively (Figure 7C). In the presence of both the compounds, the PdsA levels decreased in AX2 cells suggesting that either the cAMP relay becomes weaker or pulsing time gets longer than the expected 6 minutes. Caffeine indirectly might rescue the *pde4*^- ^phenotype by inducing the over expression of other extracellular cAMP phosphodiesterase (PdsA).

### Decreasing extracellular cAMP levels increases the aggregate size

**Figure 8:**
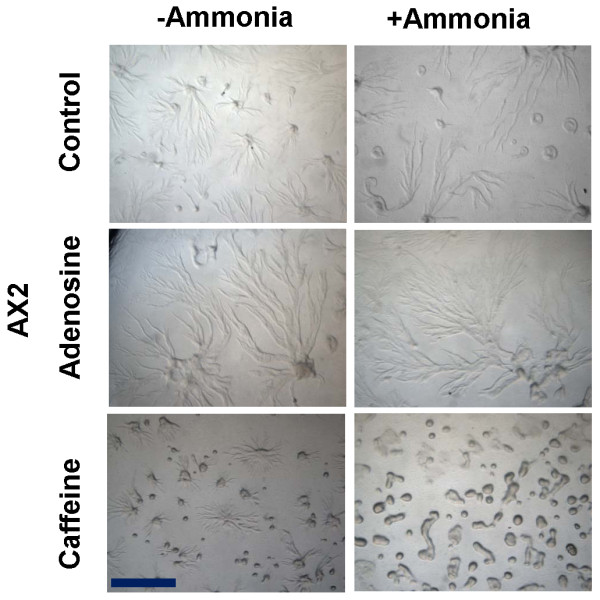
**The aggregate formed in the presence of ammonia were comparatively larger than the respective controls**. Ammonia was generated by mixing 2 ml of 2 mM NH4Cl solution and 2 ml of 1N NaOH solution [38]. A total of 4 ml solution was poured on the top lid and the dishes having the cells (plated on agar media) were kept inverted. To prevent the diffusion of gas, the plates were tightly sealed with the parafilm. Scale bar = 1000 μm.

It is known that addition of extracellular cAMP to *Dictyostelium *cells reduces the aggregate size [47]. Ammonia, one of the mophogenetic regulators in *Dictyostelium *is known to reduce extracellular cAMP levels [47]. We used ammonia to reduce the extracellular cAMP levels and in the same environment, the cells were allowed to develop on adenosine containing plates to monitor the aggregate size. Aggregates formed in the presence of ammonia (control) were large (Figure 8) but when aggregation took place in the presence of ammonia and adenosine, the sizes were even larger compared to aggregates formed in the presence of ammonia (Figure 8). The aggregates formed in the presence of caffeine together with ammonia were smaller compared to aggregates formed in the presence of caffeine alone. This result suggests that adenosine might be increasing the aggregates size by reducing the extracellular cAMP levels and also the ratio of cAMP/adenosine may be a significant factor in determining aggregate size. Caffeine and ammonia together by inhibiting cAMP relay might be enhancing cytosolic glucose levels and cell adhesion protein expression thereby forming streamless compact aggregates.

### Adenosine slows cell motility and caffeine enhances it

**Figure 9:**
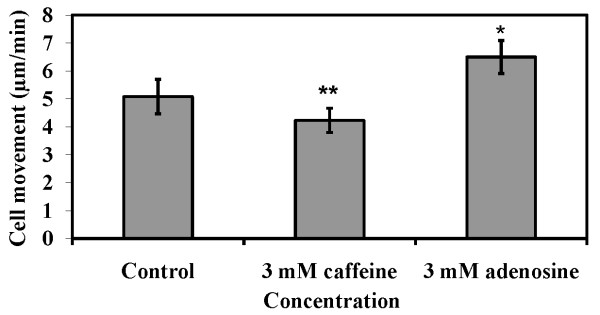
**Cell movement of AX2 cells in the presence of 2 mM caffeine or 2 mM adenosine**. Cell movement assay was performed as described in materials and methods. The values represent mean ± standard deviation from 54 cells obtained from three independent experiments. The significance of experiment was checked performing Student's t-test (*P < 0.03, **P < 0.05).

If the cAMP relay becomes weak or its pulsing is delayed, the motility of cells could be affected [43]. If there is rapid movement of the amoebae towards the chemotactic centre, they might end up in forming several smaller-sized aggregates than cells that move slowly [41]. In the presence of 2 mM adenosine, the cell speed was about 4.22 ± 0.43 μm/min (p < 0.05) whereas in 2 mM caffeine, the movement accelerated to about 6.49 ± 0.59 μm/min (p < 0.03). In absence of these compounds, cell speed was 5.07 ± 0.61 μm/min (Figure 9). Thus, the presence of adenosine decreased cell motility while the presence of caffeine increased cell motility. This decrease in cell motility might influence the amoebae to join the existing aggregates rather than to form a new one. The average speed of the chemotactic amoebae towards the aggregation centres either with caffeine or adenosine could be one of the factors that determining aggregate size.

### Adenosine and caffeine regulate aggregate size by altering the counting mechanism

**Figure 10:**
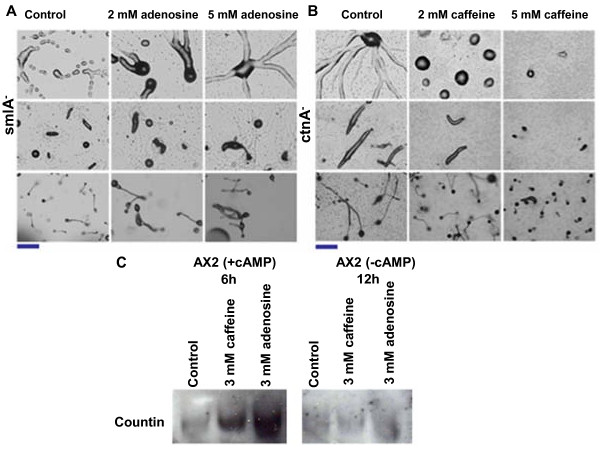
**A) Effect of adenosine on *smlA^- ^*cells: Adenosine prevented the breaking of the aggregation stream leading to formation of big sized slugs and fruiting bodies**. B) Effect of caffeine on countin (c*tnA^-^*) null cells: With increasing concentration of caffeine, the size of the aggregate, slug, and fruiting body gets reduced. Scale bar = 200 μm. C) Western blots of Countin in the presence or absence of either caffeine or adenosine.

The aggregate size defective mutant *smlA *of *D. discoideum *forms small aggregates and slugs, and finally small fruiting bodies [13]. The *smlA *gene encodes a protein, which appears to regulate the secretion of the counting factor (CF) that in turn regulates the aggregate size [25]. To check if adenosine can alter the aggregate size of this mutant, the amoebae were allowed develop on non-nutrient medium containing various concentrations of adenosine or caffeine. By increasing adenosine concentrations, the aggregate size increased gradually and at 5 mM adenosine, the aggregate size was comparable to that of the parental controls (Figure 10A). Surprisingly, caffeine showed insensitive response to aggregation and there was no change in size of the aggregates and fruting bodies (Additional file 6, Fig S5). Another aggregate size defective mutant, *ctnA *(*countin*), forms large aggregates, elongated slugs, and large fruiting bodies [10,13,26]. Countin belongs to the counting factor complex, which partially regulates aggregate size [25]. We therefore wanted to verify whether the size of *ctnA*^-^null mutant aggregates can be altered by adenosine and caffeine. There was a correspondence of increasing caffeine concentrations to reduced aggregate size in these mutants (Figure 10B). The large aggregates and fruiting bodies formed by *ctnA*^- ^mutants became progressively smaller with increasing caffeine concentrations (Figure 10B) and progressively got larger with increasing adenosine concentrations (Additional file 7, Fig S6). Thus, both adenosine and caffeine override the impact of mutations in SmlA and CntA genes on aggregate size, respectively.

*smlA^- ^*cells overexpress counting factors (CF) and forms small aggregates and mutants with impaired countin expression favours large aggregation territories. The small aggregates of AX2 cells in the presence of caffeine could possibly be due to overexpression of countin factors. We monitored the expression of countin by western blot analysis in vegetative AX2 cells pulsed with cAMP and cells not pulsed with cAMP. In pulsed control cells without adenosine or caffeine, Countin expression at 6 hours of development was barely detectable whereas in unpulsed cells developed for 12 hours, no sign of Countin expression was observed either (Figure 10B). In the presence of 3 mM adenosine or caffeine, pulsed cells at 6 hours showed an increase of Countin expression while both compounds could not induce the expression of countin in unpulsed cells at 12 hours (Figure 10C). Though ccompared to the two mutants, *smlA^- ^*and *ctnA^- ^*wild type cells may have enhanced countin expression, it is interesting to note that *ctnA^- ^*also forms small aggregates in the presence of caffeine. This suggests that other Countin factors such as CF45-1; CF-50; CF-60 may be involved in regulating the aggregate size in this mutant in the presence of caffeine. On the other hand, adenosine overcomes the *smlA^- ^*defect in forming large aggregates. Though there was increased countin expression in the presence of adenosine, still large aggregates were formed and this may be independent of counting mechanism.

## Discussion

The total cell number and their individual size contribute to the size of a tissue, organ, or an organism [12]. In *D. discoideum*, four prominent factors are known to affect the aggregate size, namely, cell number and cell volume, cell density sensing countin factors, cell adhesion and cAMP relay. In this study, we examined all the factors that contribute to a change in aggregate size in the presence of adenosine or caffeine as either one of them changes the size of aggregates. Interestingly, few responses to caffeine and adenosine were opposite to each other (cell proliferation rates, cell size, rates of cell movement) while other responses were similar (cAMP signal relay, cell adhesion and countin expression). However, the aggregate sizes were always large with adenosine and small in the presence of caffeine.

### Cell number and cell size

There was an opposing effect of adenosine and caffeine on cell division rates and cell size. The amoebae became smaller with caffeine and the cell doubling time got longer than the controls. On the other hand, adenosine increased the growth rates thereby increasing the cell number and it also increased the cell size. Caffeine is an inhibitor of Target of Rapamycin (TORC1), and yeast cells lacking Tor1p, a component of TORC1, are resistant to caffeine [48]. The TORC1 complex is a major pathway that determines cell growth and cell size [14]. In nutrient poor conditions or in glucose depleted conditions, the TORC1 complex is inactivated [14], leading among other downstream effects to autophagy induction [49]. The TORC2 complex maintains cell integrity, cell morphology, chemotaxis and cell polarity [50,51]. Based on the observations in yeast and other organisms, it is likely that caffeine induced reduction in cell number and cell size may be mediated by down regulating TORC1 activity in *Dictyostelium *also [52]. Adenosine might be enhancing the TORC1 activity favouring an increase in cell number and cell size. In yeast, caffeine is also known to affect DNA repair mechanisms and inhibits cell cycle progression [53]. Both the compounds may be targeting the genes involved in regulating cell proliferation thus altering the cell number in *Dictyostelium *also. Small aggregates formed in the presence of caffeine may be due to fewer cells available for aggregation and the cell sizes were also smaller compared to controls. Contrastingly, if there is an increase in cell number and an increase in cell size that would significantly contribute to enlarged aggregate size as with adenosine. Adenosine is likely to contribute additional cell divisions beyond starvation thereby making more cells available in a defined area for aggregation compared to controls and caffeine treated cells. Caffeine's effect seems to be pleiotropic and may depend on cell cycle stages. Caffeine might affect a fraction of the cells to have delayed cell division while in others it may completely stop cell division and relay, and this would result in a number of single cells without participating in aggregation as observed in caffeine containing plates.

### Relayed cAMP signal

During development, pulses of extracellular cAMP attract the amoebae located farther apart towards the aggregation centres or the territories. We monitored aggregate pattern and size of the cells with mutations in the key enzymes in cAMP relay that regulate synthesis (ACA), and degradation of extracellular cAMP. *acaA*^- ^cells do not make cAMP, so cells remain unicellular without aggregating during starvation [54-56]. In *acaA*^- ^cells, neither caffeine nor adenosine induced aggregation suggesting that both compounds do not activate other adenyl cyclases (ACB and ACG) for rescuing the defective aggregation phenotype. In wild type cells, caffeine might enhance PDI activity while down regulating PdsA levels and if that happens, then it would favour small aggregate formation. However, caffeine induced the formation of large aggregate territories in *pdiA^- ^*and *pde4^- ^*mutants. *PdiA*^- ^mutants form aggregates with no streams but the streaming defect could be rescued in the presence of caffeine or adenosine. This suggests that the cAMP phosphodiestrease levels might have increased in the presence of these two compounds resulting in decreased cAMP levels in the extracellular environment that is responsible for large aggregation territory sizes. *pde4*^- ^cells do not stream and forms small clumps of cells. By activating other phosphodiesterases or proteins similar pdiA, caffeine might possibly rescue the function of these mutants that have defective aggregation. Unlike wild type cells, in *pde4^- ^*cells there was enhanced expression of PdsA in the presence of caffeine and adenosine. The large aggregation stream and increased expression of PdsA in *pde4^- ^*cells in the presence of caffeine suggests reduced cAMP accumulation in extracellular media. These results with adenosine and caffeine on mutants impaired in cAMP relay suggest that cAMP level is a major factor contributing to changes in aggregate sizes. Reconstitution of wild type cells with *acaA*^- ^cells rescue aggregation defect in controls while in the presence of caffeine or adenosine, there were large number of aggregates implying that cAMP degradation is prevented by both these compounds. This was indirectly corroborated by western blotting showing that expression of extracellular cAMP phosphodiesterases is reduced significantly in wild type cells in the presence of caffeine or adenosine. Adding extracellular cAMP resulted in small aggregate formation [47] and decreasing the levels of cAMP by ammonia increased the aggregate size (Figure 8) suggesting, extracellular cAMP levels play a crucial role in determining the streaming, patterning and territory size. It is also likely that the ratio of cAMP to adenosine determining the aggregate size in *Dictyostelium*.

During development, caffeine is known to inhibit adenyl cyclase-A (ACA) activation [34] thereby delaying the cAMP relay and weakening the signal strength. These all contribute to affect the timing of aggregate formation. It is known that in the constant presence of caffeine, cAMP relay gets affected [34] which might be responsible for small aggregate formation.

Though the strength of cAMP relay gets weak with both the compounds, the differential directional motility of cells could still be a factor determining the aggregate size [57]. It is known that in conditions of enhanced cell movement small sized aggregates were formed [41]. Thus it is likely that a weak cAMP relay and increased cell motility in the presence of caffeine favouring the formation of many small aggregates. Although the strength of cAMP is weak in the presence of adenosine, the amoebae move far slowly which might influence the amoebae to join the existing aggregates rather than to form a new one thereby forming large aggregation territories.

### Cell-cell adhesion

The integrity of the aggregates is mainly maintained by the cell adhesion protein Cad-1 and CsaA and high intracellular glucose concentrations increase cell adhesion in *D. discoideum *[10,26]. Glucose 6-phosphatase and glucokinase are the key enzymes regulating internal glucose levels by reversibly converting glucose-6-phosphate into glucose and vice versa [58]. Supplementing glucose increases cytosolic glucose levels and cell-adhesion protein expression thereby favouring large aggregate size. Both adenosine and caffeine increased cytosolic glucose levels and cell adhesion protein expression causing a strong cell-cell adhesion. However, the altered aggregate sizes that we observed in the presence of these two compounds may not be due to cell adhesion protein expression alone as caffeine, similar to adenosine increased cell adhesion via increased expression of cell adhesion protein. The small but tight aggregate formed in the presence of caffeine may require strong adhesion so that the integrity is not lost and this is supported by enhanced CsaA expression and not Cad-1 (Figure 5B). Maintaining the large aggregate size may also require high expression levels of both CsaA and Cad-1. Adenosine strongly induced the expression of both the cell adhesion proteins. Aggregates formed in the presence of adenosine formed large streams which may require the both CsaA and Cad-1 expression while compact aggregates formed with caffeine had little streaming and this might have caused the expression of CsaA alone. High expression levels of the cell adhesion proteins CsaA and Cad-1is likely to contribute to changes in aggregate sizes. Adenosine may be particularly exerting its effect on cell adhesion during stream formation.

In 1986, Hangmaan [59] had shown that cells grown in medium containing caffeine develop more rapidly (2 hours earlier) when developed in a non-nutrient medium lacking caffeine, which is likely by inducing overexpression of adenyl cyclase-A. Amoebae grown with caffeine and developed in its absence formed large aggregates with normal streaming, and in these conditions, adenyl cylacse-A activity may be high. However, caffeine during development may be inhibiting adenyl cyclase activity favouring small aggregate formation with little streaming. When adenyl cyclase-A expression is high large aggregates were formed and when the expression is low small aggregates were formed. Caffeine, increased intracellular glucose levels which might have an influence on the aggregate size as high glucose levels are known to increase the aggregate size. However, glucose levels were still high when cells were developed in the presence of caffeine and aggregates were still small. During starvation in the presence of caffeine, factors other than glucose may be deciding the aggregate size.

Similarly, we observed precocious development and increased expression of early differentiation markers in cells pulsed with cAMP in the presence of caffeine, which may be due activation of adenyl cyclase-A (acaA) and cAMP receptors (cAR1) (Additional file 3, Additional file 8, Fig S7, Additional file 9).

### Counting mechanism

**Figure 11:**
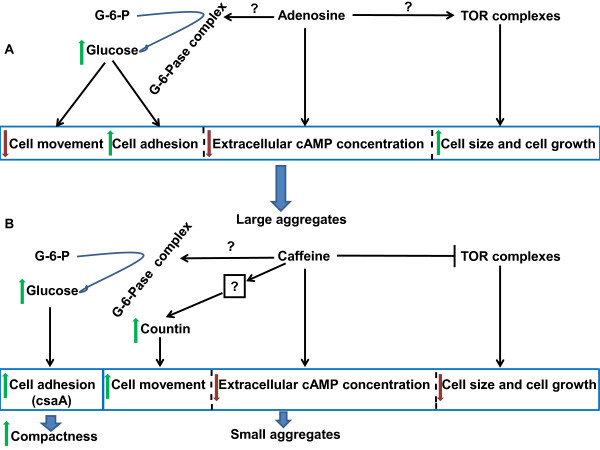
**Model of aggregate size regulation in *Dictyostelium *by adenosine and caffeine**. **A) **At the aggregation stage, adenosine may be decreasing extracellular cAMP accumulation, favouring cell growth and this might be due to enhanced TOR activity and increased cell movement and cell adhesion by increasing the cytosolic glucose concentration. Collectively all these parameters cause large aggregate formation in the presence of adenosine. **B) **Inactivation of TOR complex by caffeine leads to reduction in cell number and cell size. Caffeine increases countin expression acting on unknown upstream targets resulting in increased cell movement. Low levels of extracellar cAMP, faster cell movement and few cells available for aggregation are plausible reasons for small sized aggregate formation with caffeine. The compactness of the aggregate is maintained by expression of cell adhesion protein CsaA mainly.

In *D. discoideum*, the secreted counting factor complex (CF) maintains group size by regulating cell-cell adhesion and cell motility [15,26]. Supplementing glucose mimics caffeine effect (Fig S4). The increase in intracellular cytosolic glucose levels decreases countin factor levels [11,57] and increases cell-cell adhesion. *smlA^- ^*cells forms small aggregates as a result of over expression of counting factors [25] and adenosine might restore the parental phenotype possibly by down regulating the CF expressions, increasing glucose level and cell-cell adhesion. Insensitive caffeine responses in *smlA^- ^*cells suggest caffeine does not increase the countin factor expression further. In the presence of caffeine, Countin expression in AX2 cells pulsed with cAMP was enhanced, maybe leading to small aggregate formation. In *ctnA^- ^*cells, which forms large aggregates, caffeine restores parental aggregate size by decreasing the intracellular glucose levels (unpublished data), increasing countin factors level and possibly by weakening cellular adhesion. The elevated countin expression in the presence of adenosine though favours large aggregate formation (Figure 11C) may be independent of the countin mechanism.

The four prominent factors regulating the aggregate size were mainly examined in *D.discoideum*. The conserved effect of adenosine and caffeine on the aggregates size of other slime molds suggests that other species may also be having a similar mechanism in aggregate size regulation. *Polysphondylium *also strikingly forms large and small aggregates with adenosine and caffeine an effect which was quantitated. Though extracellular chemotactic signals may be different, the intracellular pathways regulating aggregate size may be common in all these slime molds. It is interesting that a chemotactic signal molecule used for cell-cell communication is also used for regulating the multicellular tissue size. It is likely that in other slime molds using chemoattractants other than cAMP, both adenosine and caffeine may cause a defective relay as they also form large and small aggregates, respectively.

## Conclusion

In this study, we show that certain mechanisms such as the regulation of aggregate size are conserved among distantly related slime molds. One conserved phenotype is that for all studied organisms, the aggregates become larger in the presence of adenosine and smaller in the presence of caffeine suggesting that the factors/mechanism regulating aggregation sizes might also be similar in other slime molds. Adenosine increases cell size as well as cell number contributing to increased aggregate size while caffeine, reduced the cell size and number, favouring small aggregate formation. Both the compounds affect the speed of the chemotactic amoebae during aggregation causing a variation in the size of aggregates. Though, certain molecular processes induced by adenosine and caffeine are similar the aggregation phenotype was always different between these two compounds. The identical effects induced by both the compounds include increased cell adhesion protein (Cad-1) expression, high glucose levels and altered cAMP relay. The large aggregate with many streams formed in the presence of adenosine may be due to enhanced expression of the cell adhesion protein CsaA and Cad-1. The compactness of the small streamless aggregates formed in the presence of caffeine may be due increased expression of the cell adhesion protein CsaA and not Cad-1 (Figure 11). The synthesis, secretion of other chemoattractants unrelated to cAMP could possibly be also affected by these compounds in a similar way they affect cAMP synthesis, secretion and relay (Figure 11).

## Authors' contributions

PJ performed the experiments and analyzed the data and all the authors contributed to designing the experiment, analyzing the data and writing the manuscript, and. All the authors read and approved the final manuscript.

## Supplementary Material

Additional file 1 **Figure S1: The morphology of the slug (*Polysphondylium pallidum*) in the presence of adenosine and caffeine**. Adenosine favours large slug formation whereas caffeine induces emergence of many slugs from the individual aggregate. Morphology of slugs: a) Control; adenosine at (b) 0.5 mM (c) 3.5 mM and caffeine at (d) 1 mM (e) 2.5 mM (f) 3.5 mM. Scale bar = 200 μm. Click here for file

Additional file 2 **Figure S2: The effect of adenosine deaminase on aggregation**. **A) **Aggregation pattern: Adenosine deaminase induced formation of several small sized aggregates. We treated PN500 cells with 50 unit/ml of adenosine deaminase for 2 hours and subsequently plated on non-nutrient agar surface (absence of adenosine deaminase). Aggregation was observed under the light microscope. B) Time of aggregation: Adenosine deaminase delayed the aggregation for the 4 hours. C) Number of aggregates were formed with adenosine deaminase were more than control (absence of it) (Student's t-test, *p < 0.001). Click here for file

Additional file 3 **Effect of caffeine and adenosine on streaming and aggregation pattern**. Aggregation pattern and expression analysis of early gene diffrentiation are mentioned. Click here for file

Additional file 4 **Figure S3: Aggregation pattern and streaming of AX2 cells in starvation buffer in the presence of caffeine and adenosine**. Both adenosine and caffeine inhibited spiral wave formation in aggregates. The arrow sign in 2^nd ^lane of Figure 2D indicates arrangement of the cells at aggregation centres. The aggregates were developed in 90 mm petri dish submerged with Sorensen buffer containing either 3 mM adenosine or 3 mM caffeine. Click here for file

Additional file 5 **Figure S4: Cytosolic glucose levels of developing cells in the presence or absence of 5 mM glucose**. The glucose level was estimated as described in materials and methods. The values represent mean ± standard deviation (n = 6), *P < 0.001 (Student's t-test). Click here for file

Additional file 6 **Figure S5: A) The Effect of 2 mM and 5 mM caffeine on the aggregates and fruiting bodies of AX2 cells**. B) Effect of 2 mM and 5 mM caffeine on the aggregates and fruiting bodies of smlA mutant cells. Scale bar = 200 μm. Click here for file

Additional file 7 **Figure S6: A) Effect of 2 mM and 5 mM adenosine of the aggregates and fruiting bodies of AX2 cells**. B) Effect of 2 mM and 5 mM adenosine of the aggregates and fruiting bodies of ctnA mutant cells. Scale bar = 200 μm. Click here for file

Additional file 8 **Figure S7: Caffeine shown early gene differentiation in cells pulsed with cAMP**. A) AX2 cells were incubated at a density of 1 × 10^7 ^cells/ml in the Sorensen buffer containing either adenosine or caffeine and were pulsed with 30 nM cAMP for 5 hours for every six minutes intervals. The cAMP treated cells were developed on non nutrient agar plate in the presence or absence of either adenosine or caffeine. B) We checked the expression of acaA and cAR1 genes of pulsed AX2 cells in the presence or absence of either caffeine or adenosine by performing qRT-PCR. The values represent mean ± standard deviation from three independent experiments. C) Cell adhesion protein (Cad-1 and CsaA) and the early gene induction marker protein (CsaA) expressions: To check expression levels, we performed Western blot of these proteins in AX2 cells pulsed with 30 nM cAMP in the presence or absence of adenosine or caffeine. Click here for file

Additional file 9 **Description of Quantitative Reverse Transcription-Polymerase Chain Reaction (qRT-PCR)**. Method and primer sequences used in this study are mentioned [60]. Click here for file
