# Supplementary figures and images for: Regulation of aggregate size and pattern by adenosine and caffeine in cellular slime molds
Source: BMC Dev Biol. 2012 Jan 23;12:5. doi: 10.1186/1471-213X-12-5 (PMC3341216; doi:10.1186/1471-213X-12-5)

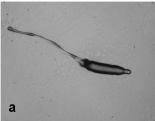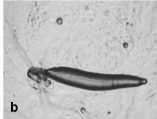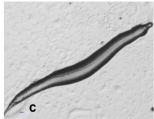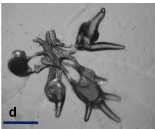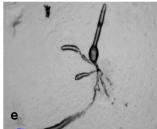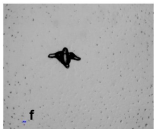

Supplement: Additional file 1 — Figure S1: The morphology of the slug (Polysphondylium pallidum) in the presence of adenosine and caffeine. Adenosine favours large slug formation whereas caffeine induces emergence of many slugs from the individual aggregate. Morphology of slugs: a) Control; adenosine at (b) 0.5 mM (c) 3.5 mM and caffeine at (d) 1 mM (e) 2.5 mM (f) 3.5 mM. Scale bar = 200 μm. [file 1471-213X-12-5-S1.PDF]

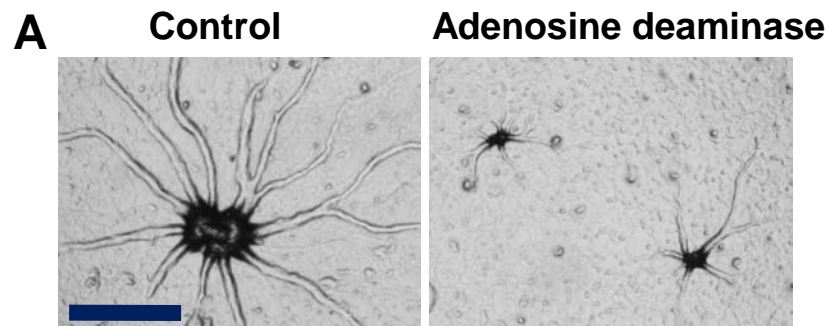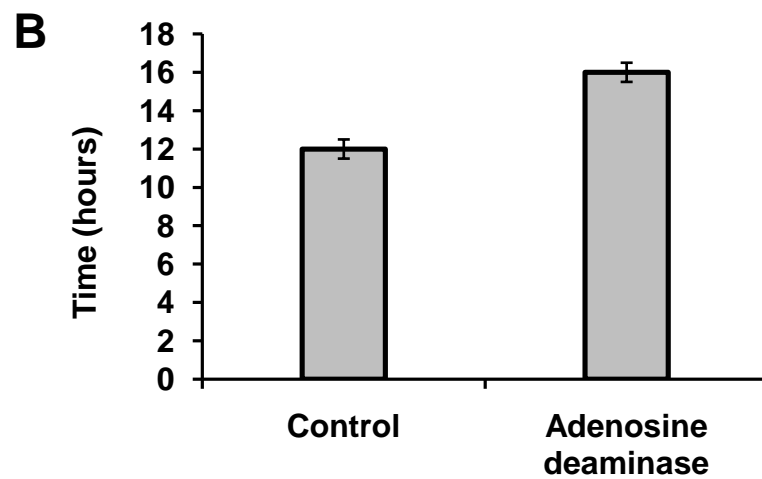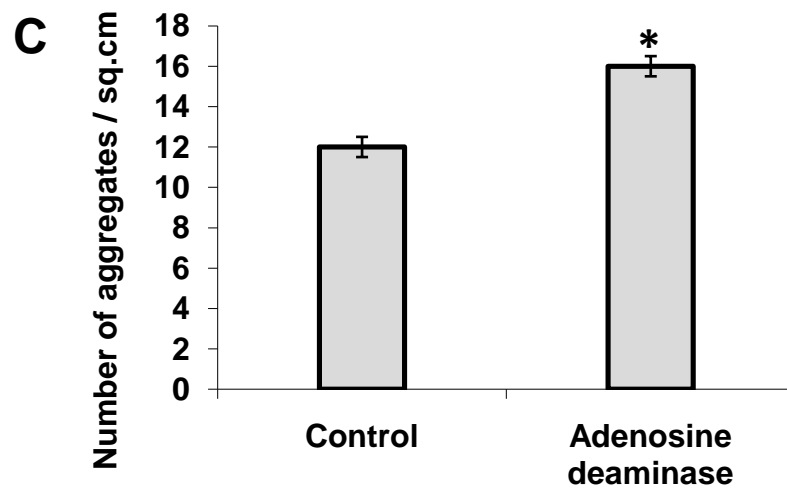

Supplement: Additional file 2 — Figure S2: The effect of adenosine deaminase on aggregation. A) Aggregation pattern: Adenosine deaminase induced formation of several small sized aggregates. We treated PN500 cells with 50 unit/ml of adenosine deaminase for 2 hours and subsequently plated on non-nutrient agar surface (absence of adenosine deaminase). Aggregation was observed under the light microscope. B) Time of aggregation: Adenosine deaminase delayed the aggregation for the 4 hours. C) Number of aggregates were formed with adenosine deaminase were more than control (absence of it) (Student's t-test, *p < 0.001). [file 1471-213X-12-5-S2.PDF]

Control

3 mM adenosine

3 mM caffeine

AX2

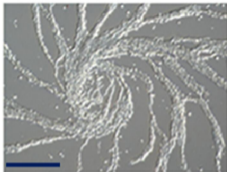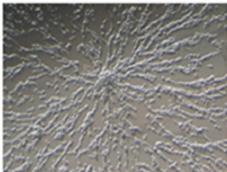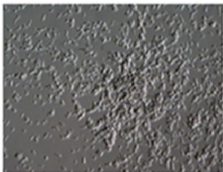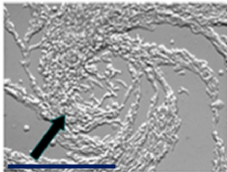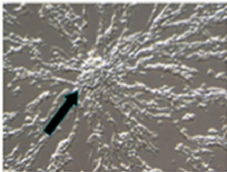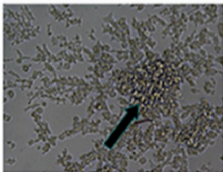

Supplement: Additional file 4 — Figure S3: Aggregation pattern and streaming of AX2 cells in starvation buffer in the presence of caffeine and adenosine. Both adenosine and caffeine inhibited spiral wave formation in aggregates. The arrow sign in 2nd lane of Figure 2D indicates arrangement of the cells at aggregation centres. The aggregates were developed in 90 mm petri dish submerged with Sorensen buffer containing either 3 mM adenosine or 3 mM caffeine. [file 1471-213X-12-5-S4.PDF]

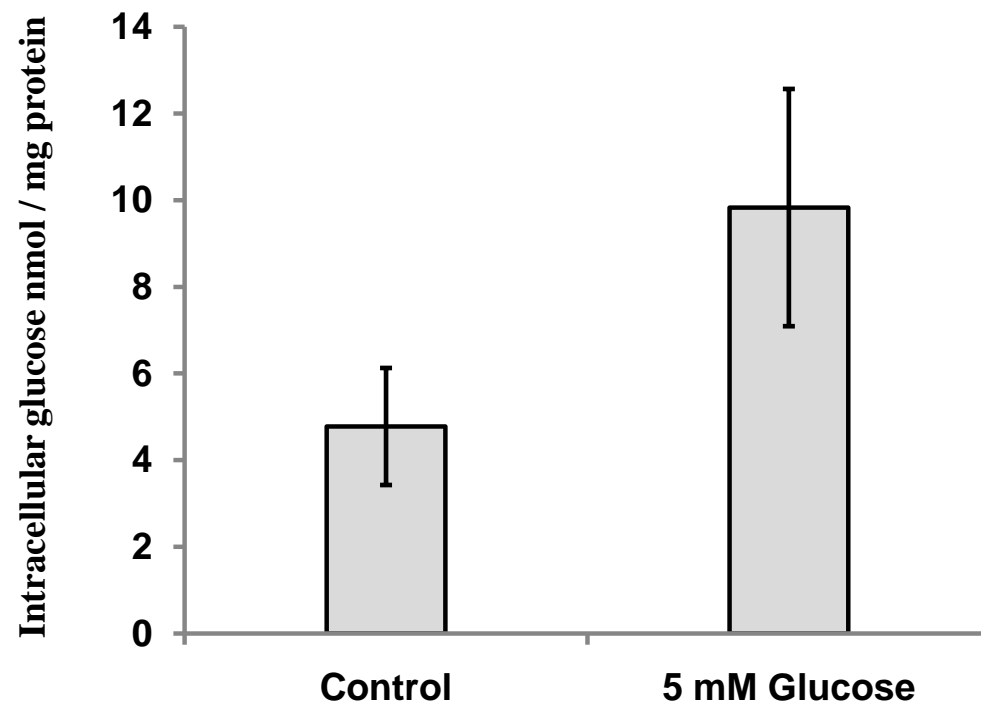

Supplement: Additional file 5 — Figure S4: Cytosolic glucose levels of developing cells in the presence or absence of 5 mM glucose. The glucose level was estimated as described in materials and methods. The values represent mean ± standard deviation (n = 6), *P < 0.001 (Student's t-test). [file 1471-213X-12-5-S5.PDF]

**A****Control****2 mM caffeine****5 mM caffeine****AX2**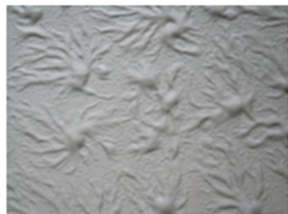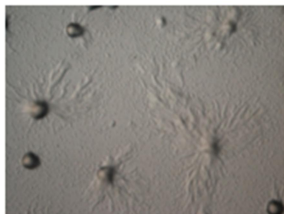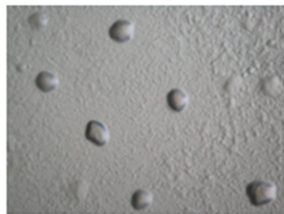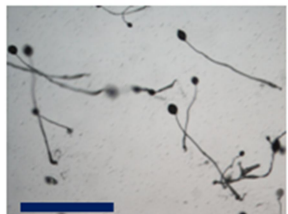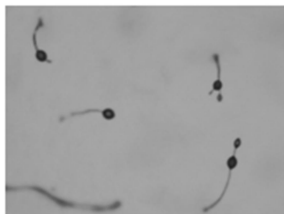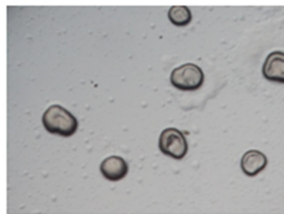**B****Control****2 mM caffeine****5 mM caffeine****smIA<sup>-</sup>**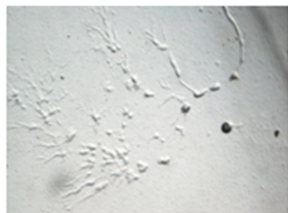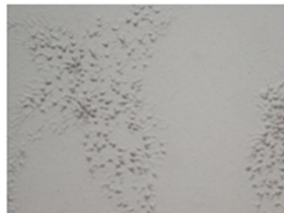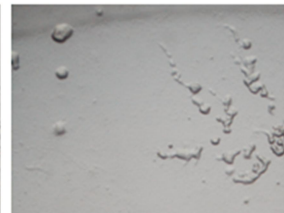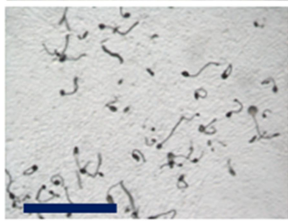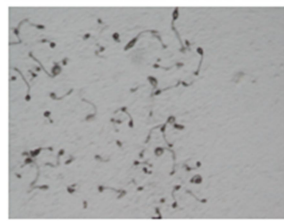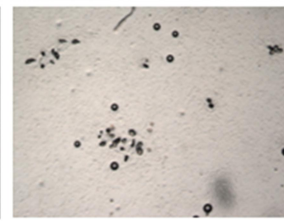

Supplement: Additional file 6 — Figure S5: A) The Effect of 2 mM and 5 mM caffeine on the aggregates and fruiting bodies of AX2 cells. B) Effect of 2 mM and 5 mM caffeine on the aggregates and fruiting bodies of smlA mutant cells. Scale bar = 200 μm. [file 1471-213X-12-5-S6.PDF]

**A****Control****2 mM adenosine****5 mM adenosine****AX2**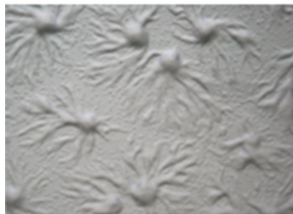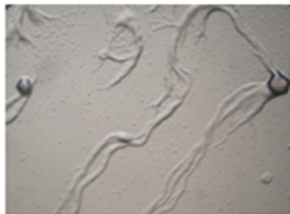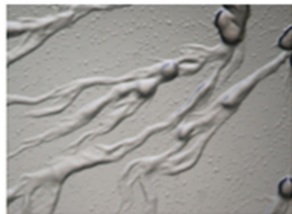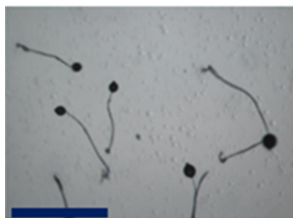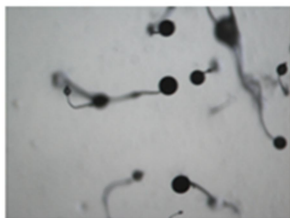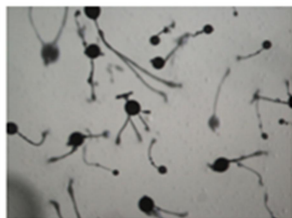**B****Control****2 mM adenosine****5 mM adenosine****ctnA<sup>-</sup>**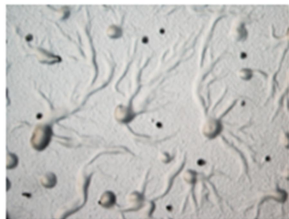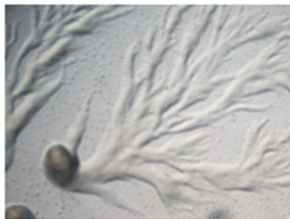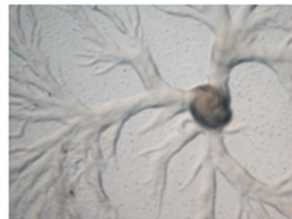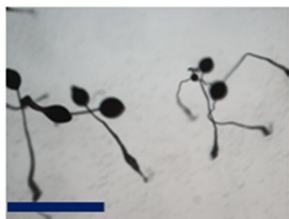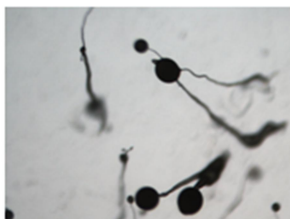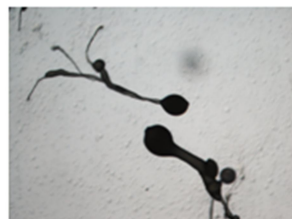

Supplement: Additional file 7 — Figure S6: A) Effect of 2 mM and 5 mM adenosine of the aggregates and fruiting bodies of AX2 cells. B) Effect of 2 mM and 5 mM adenosine of the aggregates and fruiting bodies of ctnA mutant cells. Scale bar = 200 μm. [file 1471-213X-12-5-S7.PDF]

**A**

|                |   |   |   |   |   |   |
|----------------|---|---|---|---|---|---|
| cAMP           | - | + | + | + | + | + |
| Adenosine      | - | - | + | + | - | - |
| Caffeine       | - | - | - | - | + | + |
| KK2+ caffeine  | - | - | - | - | - | + |
| KK2+ adenosine | - | - | - | + | - | - |

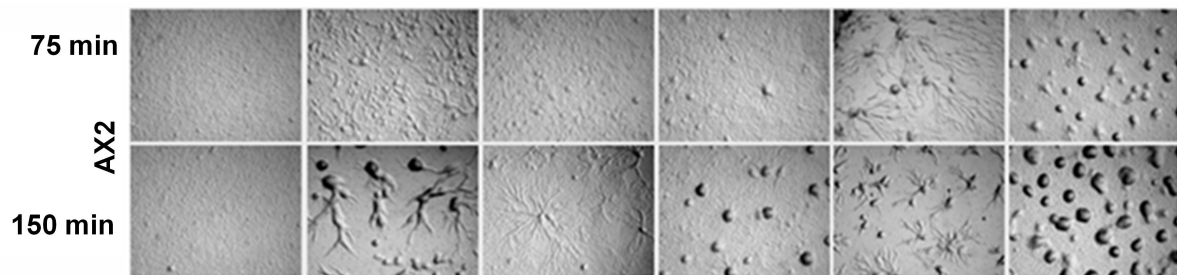**B**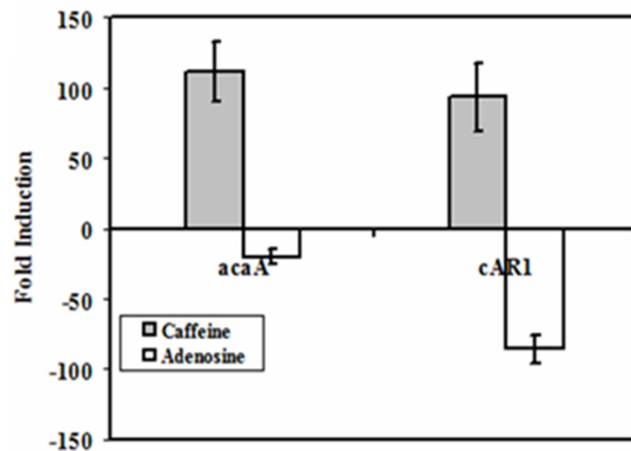**C**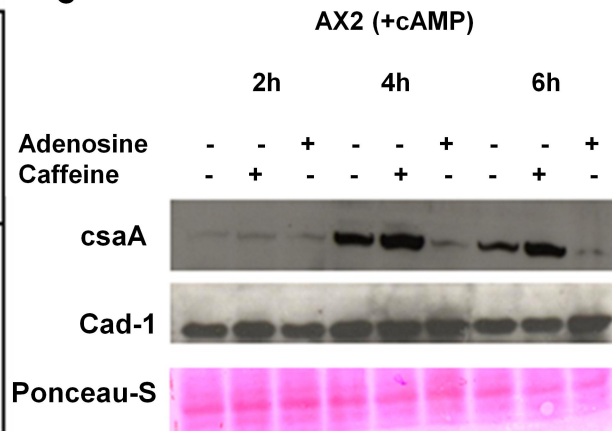

Supplement: Additional file 8 — Figure S7: Caffeine shown early gene differentiation in cells pulsed with cAMP. A) AX2 cells were incubated at a density of 1 × 10^7^ cells/ml in the Sorensen buffer containing either adenosine or caffeine and were pulsed with 30 nM cAMP for 5 hours for every six minutes intervals. The cAMP treated cells were developed on non nutrient agar plate in the presence or absence of either adenosine or caffeine. B) We checked the expression of acaA and cAR1 genes of pulsed AX2 cells in the presence or absence of either caffeine or adenosine by performing qRT-PCR. The values represent mean ± standard deviation from three independent experiments. C) Cell adhesion protein (Cad-1 and CsaA) and the early gene induction marker protein (CsaA) expressions: To check expression levels, we performed Western blot of these proteins in AX2 cells pulsed with 30 nM cAMP in the presence or absence of adenosine or caffeine. [file 1471-213X-12-5-S8.PDF]
