# Supplementary material for: Regulation of aggregate size and pattern by adenosine and caffeine in cellular slime molds
Source: BMC Dev Biol. 2012 Jan 23;12:5. doi: 10.1186/1471-213X-12-5 (PMC3341216; doi:10.1186/1471-213X-12-5)
Supplement: Additional file 3 — Effect of caffeine and adenosine on streaming and aggregation pattern. Aggregation pattern and expression analysis of early gene diffrentiation are mentioned. [file 1471-213X-12-5-S3.DOC]

**Adenosine and caffeine perturbs the formation of aggregate centers in *Dictyostelium*.**

Adenosine is known to induce formation of large aggregates in *D. discoideum* [30] but its effect on pattern formation during aggregation is not known. We developed aggregates of AX2 cells on starvation plates (Sorensen buffer) containing the desired concentrations of adenosine or caffeine. There was a 4 hour delay in the formation of the aggregation centres with both adenosine and caffeine. In controls, the aggregation spirals were evident and in the presence of these two compounds, there were no spirals suggesting that both adenosine and caffeine perturbing spiral wave formation and cAMP relay. Adenosine favoured thin but large aggregation territories and cells appear firmly adhered to each other (Additional file 4, Fig S3). As development progresses, the slender stream move toward the aggregation centre and gets compact (Additional file 4, Fig S3). However, many stream-less but small aggregates were formed in the presence of 3 mM caffeine (Additional file 4, Fig S3). Thus, if the stream extends its territory in the presence of adenosine, then aggregate size would also go up correspondingly. There was no stream formation in the presence of caffeine and that could also contribute to small aggregate formation.

**Caffeine induces expression of early differentiation genes in cells pulsed with cAMP.**

As shown in Fig. 7C, both caffeine and adenosine affect the expression levels of the cAMP phosphodiesterase PdsA. On the other hand, it is known that cells grown in the presence of caffeine increases adenyl cyclase activity by 30 folds [34, 54, 59]. If the PdsA expression levels are low, then cAMP receptors and adenyl cyclase enzyme may be activated and the cAMP relay signal might be stronger. To verify if amoebae pulsed in the presence of caffeine or adenosine differentiates earlier we monitored aggregation of AX2 cells pulsed with cAMP in presence or absence of either caffeine or adenosine. Pulsed cells streamed 75 min after plating on non-nutrient agar, while signs of aggregation could be seen in unpulsed cells only after 150 min subsequent to plating (Additional file 8, Fig S7A). After cAMP pulsing, adenosine did not affect the streaming, and aggregates were observed at the same time as in the absence of adenosine. In contrast, cells pulsed in the presence of caffeine formed aggregates earlier than controls, independently of whether caffeine was added only during pulsing or if caffeine was also added into the agar. After cAMP pulsing, presence of caffeine induced early aggregation, but in the absence of pulsing, there was a delay in aggregate formation.

If the cAMP receptor cAR1 is activated, then Gβγ subunits will activate adenyl cyclase (acaA) to catalyze the conversion of ATP into cAMP. To monitor whether caffeine or adenosine alters expression of cAR1 or the adenyl cyclase AcaA, we perfomed qRT-PCR in AX2 cells pulsed with cAMP in presence or absence of the compounds. Interestingly, expression of cAR1 and acaA were up-regulated by 111 ± 21.27 and 93.6 ± 23.73 folds, (Additional file 8, Fig S7B).) respectively in the presence of caffeine. The expression of both genes was down regulated in the presence of adenosine, down by 86.0 ± 10.0 for cAR1 and by 20.0 ± 5.0 folds (Additional file 8, Fig S7B), for acaA. This suggests that early differentiation genes cAR1 and AcaA are activated in cells pulsed with cAMP in the presence of caffeine.

Further, we performed western blot for early differentiation markers (CsaA, Cad-1) in AX2 cells pulsed with cAMP in presence or absence of adenosine or caffeine (Additional file 8, Fig S7C). These proteins expressed earlier in cells pulsed in the presence of caffeine and there was a two fold increase in the expression of CsaA and Cad-1. However, in the presence of adenosine, CsaA was almost undetectable whereas Cad-1 expression decreased up to two-fold. Taken together, the data suggest that caffeine induces early differentiation in cells pulsed with cAMP by inducing cAMP relay signal and expression of early differentiation markers.
