# Supplementary material for: Regulation of aggregate size and pattern by adenosine and caffeine in cellular slime molds
Source: BMC Dev Biol. 2012 Jan 23;12:5. doi: 10.1186/1471-213X-12-5 (PMC3341216; doi:10.1186/1471-213X-12-5)
Supplement: Additional file 9 — Description of Quantitative Reverse Transcription-Polymerase Chain Reaction (qRT-PCR). Method and primer sequences used in this study are mentioned [60]. [file 1471-213X-12-5-S9.DOC]

**Methods**

**Quantitative Reverse Transcription-Polymerase Chain Reaction (qRT-PCR)**

We performed qRT-PCR to check the expression levels of acaA and cAR1 mRNA. AX2 cells, grown in HL5 medium were harvested and developed (in Sorensen buffer in the presence or absence of either adenosine or caffeine) with 30 nM cAMP pulses in every six minutes intervals for a period of 5 hours. Using a Qiagen RNeasy mini kit, RNA was isolated from 1X 10^7^ cells and its integrity was checked in a 1% formaldehyde agarose gel. With using random primers, cDNA was prepared using Go Script^TM^ Reverse Transcription system (Promega-USA). The primer sequences are as follows [60]: CarA-1 forward-ATGTTGGGTTGTATGGCAGTG, CarA-1 reverse-AGGGAAACCACCATTGACAG; acaA forward-CATTCTAGAGGCGGTATTGGC, acaA reverse-GGAGAAAATGTCTGATTTCGCTT; Ig7 (house keeping genes) forward- TCCAAGAGGAAGAGGAGAACTGC, Ig7 reverse-TGGGGAGGTCGTTACACCATTC. cDNA was mixed with SYBR green and qPCR master mix (Promega-USA) and qRT-PCR was performed using 7500 applied biosystem Real Time-PCR machine.
